# *XIST* RNA: a window into the broader role of RNA in nuclear chromosome architecture

**DOI:** 10.1098/rstb.2016.0360

**Published:** 2017-09-25

**Authors:** K. M. Creamer, J. B. Lawrence

**Affiliations:** Department of Neurology and Pediatrics, University of Massachusetts Medical School, Worcester, MA 01655, USA

**Keywords:** *XIST*, X-inactivation, SAF-A, nuclear matrix, nuclear scaffold

## Abstract

*XIST* RNA triggers the transformation of an active X chromosome into a condensed, inactive Barr body and therefore provides a unique window into transitions of higher-order chromosome architecture. Despite recent progress, how *XIST* RNA localizes and interacts with the X chromosome remains poorly understood. Genetic engineering of *XIST* into a trisomic autosome demonstrates remarkable capacity of *XIST* RNA to localize and comprehensively silence that autosome. Thus, *XIST* does not require X chromosome-specific sequences but operates on mechanisms available genome-wide. Prior results suggested *XIST* localization is controlled by attachment to the insoluble nuclear scaffold. Our recent work affirms that scaffold attachment factor A (SAF-A) is involved in anchoring *XIST*, but argues against the view that SAF-A provides a unimolecular bridge between RNA and the chromosome. Rather, we suggest that a complex meshwork of architectural proteins interact with *XIST* RNA. Parallel work studying the territory of actively transcribed chromosomes suggests that repeat-rich RNA ‘coats’ euchromatin and may impact chromosome architecture in a manner opposite of *XIST*. A model is discussed whereby RNA may not just recruit histone modifications, but more directly impact higher-order chromatin condensation via interaction with architectural proteins of the nucleus.

This article is part of the themed issue ‘X-chromosome inactivation: a tribute to Mary Lyon’.

## 1. Introduction

**Figure 1.**
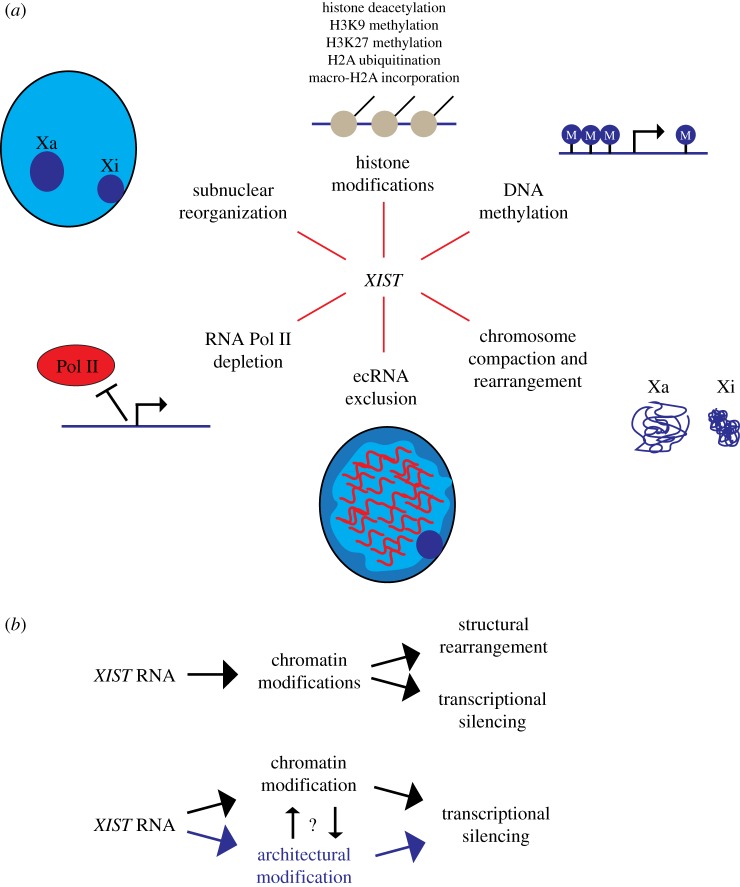
Events in X-chromosome inactivation downstream of *XIST* RNA expression. (*a*) Induction and spreading of the *XIST* RNA on the X chromosome directly or indirectly triggers a number of changes leading to robust transcriptional silencing. The timing and interplay between these events is only beginning to be understood. *XIST* is thought to directly bind and recruit one or more histone modifying enzymes which introduce histone deacetylation, methylation and ubiquitination of H2A, leading to other downstream modifications, including incorporation of histone variant macro-H2A, and CpG methylation, as well as other changes to create an environment that represses transcription. This series of numerous histone modifications coincides with large-scale changes in nuclear organization, including exclusion of repeat-rich euchromatin-associated RNAs (ecRNAs) and Pol II, chromatin compaction (visible DNA condensation), topological rearrangement of the chromosome territory, and movement of Xi to the peripheral heterochromatin compartment (or nucleolus). Which events occur first and how these changes synergize to transcriptionally repress the whole chromosome remain to be fully established. (*b*) Current models emphasize the function of *XIST* RNA in directly recruiting histone modifying enzymes which may be responsible for transcriptional silencing and cytological-scale changes in chromosome condensation and structure (upper pathway). An alternative model is outlined in the lower pathway whereby *XIST* RNA may also directly interact and impact architectural elements of non-chromatin structure (highlighted in purple), which modifies the higher-order chromatin folding to promote chromosome condensation, movement to peripheral heterochromatin, or other overall structural changes. Thus, by interacting with a distinct set of architectural proteins (such as SAF-A) *XIST* RNA could act in parallel with chromatin modifiers, and collectively these changes may induce both gene silencing and a highly stable heterochromatic structure.

Significant strides have been made in recent years to elucidate the effectors and processes both upstream and downstream of *XIST* during X chromosome inactivation (XCI) in mammalian females. The initial coating of the X-chromosome by *XIST* RNA is known to trigger a cascade of events that result in stable chromosome-wide silencing of transcription (figure 1*a*). The resulting inactive X chromosome (Xi) is depleted of RNA polymerase II, enriched with repressive histone and DNA modifications, and structurally reorganized, as reviewed in detail elsewhere [1,2]. Histone modifications in particular are thought to be central to initiating and maintaining transcriptional silencing. However, the XCI process also results in a series of ‘packaging’ changes. For instance, HiC analysis of Xi suggests *XIST* induces a bipartite chromosome structure and disrupts intrachromosomal contacts with silenced genes [3–5]. The Xi is also physically compacted into the cytologically observable Barr body and typically localized to the nuclear periphery [6,7].

To further elucidate molecular details of this process, several recent studies have employed new technological approaches to identify *XIST* RNA binding proteins: the collective list of proteins is long, and with few exceptions, there is little consensus on many putative ‘*XIST*-interactors’ [8–11]. Though some details remain elusive and will certainly be subject to future study, there is widespread agreement from these approaches that *XIST* RNA acts at least in part via directly inducing a histone modification cascade. In support of this idea, *XIST* recruitment of histone deacetylase activity by HDAC3 via interaction with the SHARP/SPEN protein is an essential step in XCI [8,9,12]. Furthermore, *XIST* has been reported to interact with subunits of the polycomb repressive complex PRC1 which induce ubiquitination of histone H2A and potentially recruits the PRC2 complex to trimethylate lysine 27 of histone H3 [8,13].

Here we detail findings which lead us to consider a distinct, but complementary model whereby *XIST* RNA acts at the level of higher-order chromatin ‘architecture’. While histone modifications generate a histone ‘code’ on the 10 nm chromatin fibre, we use the term ‘architecture’ here to refer to elements that influence the higher-order folding of that chromatin fibre. It may be widely assumed that visible condensation of the inactive X chromosome into the Barr body is due to collective histone modifications or silencing transcription of genes along the chromosome. But the causal nature of this relationship may not be that straightforward. For instance, Chandra *et al*. and our laboratory recently provided evidence that changes in chromatin condensation during cellular senescence appear independent of the canonical histone modifications examined [14–16]. Furthermore, recent evidence suggests that formation of the Barr body and X-linked silencing occurs similarly in different cell types and stages during development despite differing extent and distribution of underlying ‘silencing’ histone modifications [17].

Certainly histone modifications contribute to XCI, but there is evidence which leads us to suggest that *XIST* RNA also could act at a more direct level to influence architectural proteins (figure 1*b*), as discussed in part elsewhere [18–20]. In this article we consider this idea in light of recent insights into the proteins required to maintain *XIST* RNA's association with the inactive X chromosome. We further highlight evidence from our laboratory that many other RNAs are embedded with interphase chromosomes and may also act through higher-order structure. Lastly, we explore the possibility that rather than an exception in RNA biology, *XIST* serves as a window into a general property of RNAs to influence the architecture of chromosomes.

## 2. Sequences of the X-chromosome: not so special?

To understand how *XIST* influences the transcriptional output of an entire chromosome, it seems important to first identify the factors required to tether *XIST* RNA to chromatin. Does the X chromosome have unique sequences that allow *XIST* to interact specifically with one chromosome? *A priori* it seemed logical that the strict localization of *XIST* RNA to Xi could involve recognition of such X-specific sequences. It has been surprising then that the DNA sequences (and proteins, as discussed below) required for *XIST* RNA binding and silencing are not restricted to the X chromosome.

Numerous studies over the years have examined X;autosome translocations and the capacity for chromosome silencing in this context. Many focus on partial silencing elicited by X;autosome translocations, but this has the limitation that complete silencing of autosomal genes is often selected against [21–25]. Recent work from our laboratory provides a comprehensive analysis of autosomal silencing by *XIST* in the absence of selection by testing this in the context of autosomal trisomy. Our group successfully targeted a full-length (14 kb) *XIST* human cDNA into one of three chromosome 21s in induced pluripotent stem (iPS) cells from a Down syndrome patient [26]. *XIST* efficiently localized and silenced genes throughout the chromosome with remarkable efficiency, as shown by eight methods including analysis of hallmark histone modifications, chromosome compaction, CpG promoter methylation, and RNA FISH. Genome-wide transcriptional profiling suggested total transcriptional output from chromosome 21 was reduced close to disomic levels. The resulting ‘dosage compensation’ of trisomy 21 corrected phenotypic deficits *in vitro* [27], which has significance for translational research in Down syndrome. However, these results also yield important insight into *XIST* function. Genes that clearly escape silencing on chromosome 21 have not been found thus far (unpublished), and we are unaware of any ‘special sequences’ shared by chromosome 21 and the X chromosome to explain the robust silencing observed.

These findings establish that ‘special’ X chromosome enriched sequences are not required to support spreading and silencing by *XIST* RNA. Indeed, analysis of DNA sequences co-purified with *XIST* RNA during early stages of X-inactivation suggest *XIST* is not attracted to specific sequences, but spreads initially to regions topologically proximal to the site of *XIST* transcription [28]. This is followed by association with gene-rich regions before spreading more generally across the chromosome during maintenance of X-linked silencing in somatic cells [28,29]. It had been posited, most notably by Mary Lyon, that LINE-1 sequences may ‘boost’ the spread of *XIST* RNA across Xi [30–32]. This concept remains in question given that *XIST* RNA does not seem to show preference for LINE-1 elements in either the initiation or maintenance phases of XCI.

Although some sequence and epigenetic characteristics correlate with escape or insulation from XCI [33–36], these results collectively suggest that *XIST* RNA can spread and act on features common throughout the genome. *We conclude that *XIST* does not recognize the chromosome sequence, but somehow recognizes the underlying nuclear chromosome structure of its parent chromosome*. This is a remarkable feat, since there is no visible structural separation between intermingling chromosome territories in nuclei, even by standard electron microscopy [37]. Important questions remain as to how *XIST* RNA spreads from its site of transcription, binds, and organizes chromatin into a defined chromatin territory. Hence, to gain further insight into this fundamental biology, it is essential to understand the protein factor(s) that anchor RNA with chromosome structure.

## 3. ‘Tethering’ *XIST* RNA to chromatin: not so simple?

Even for *XIST*, the best studied nuclear large non-coding RNA (ncRNA), details of how the RNA binds and interacts with the chromatin remain largely unknown. It is now clear that one important protein involved in anchoring *XIST* to Xi is scaffold attachment factor A (SAF-A, also known as hnRNP-U) [38], which is broadly distributed in nuclei but enriched on Xi [39,40]. Hasegawa and colleagues have shown that SAF-A binds *XIST* RNA and is required for localizing *Xist* RNA to Xi in a mouse neuroblastoma cell line, Neuro 2A [41]. Since SAF-A has both DNA and RNA binding domains [38,42] and can bind *Xist* RNA, the predominant model postulates that SAF-A acts as a unimolecular bridge between *XIST* RNA and chromosomal DNA. Subsequent super-resolution imaging suggests that *XIST* and SAF-A signals sometimes overlap in ‘chain-like structures’ on Xi [43]. Curiously, it has been demonstrated that under certain conditions or with some antibodies SAF-A is difficult to detect in the Barr body, leading to speculation that Xi-enriched SAF-A is post-translationally modified in some way that obscures its recognition [43,44]. Such a modification or conformational change in SAF-A could have important implications for the unique structuration of the Barr body.

While we also have observed that SAF-A is absolutely required for *XIST* localization in Neuro 2A cells, recent work from our laboratory suggests in most cases the bridge between *XIST* and chromatin is more complex than a single molecule [45]. Consistent with other results, our study affirmed that depletion of SAF-A fully mislocalizes *Xist* RNA in mouse Neuro 2A tumour cells and impacts *XIST* expression/localization in pluripotent stem cells [8,9,46]. However, in several normal somatic cell types, *XIST* RNA remained localized 2–3 days after effective (approx. 90%) SAF-A protein depletion, including through cell divisions. Transformed cell lines showed variable effects following SAF-A knockdown, but none had so clear and pronounced an effect on *XIST* RNA localization as in the Neuro 2A tumour cell line [45]. These results suggest that, in some cell types at least, redundant or coordinating anchors exist to compensate for the loss of SAF-A to anchor *XIST* RNA to Xi.

The Nakamura laboratory published a response to these debated findings with new information [46], with which we find more consensus than it may at first appear. To test the effect of SAF-A in normal cells, Sakaguchi *et al*. [46] genetically deleted SAF-A from a MEF cell line, and state that this impacts *Xist* RNA ‘localization’. However, because it appears that *Xist* transcription (or stability) is disrupted, as the authors acknowledge, one cannot reliably evaluate whether *XIST* RNA localization (anchoring) is specifically impacted. At the same time, Sakaguchi and colleagues concur that there may be redundant anchors or cell type differences. They demonstrated that a protein related to SAF-A, hnRNPU-like-1, can compensate for SAF-A depletion [46]. Collectively, these results suggest other proteins can and do substitute for or collaborate with SAF-A when it comes to ‘anchoring’ *XIST*.

*XIST* RNA localizes strictly to its parent chromosome *in cis*, spreading from its site of transcription and somehow recognizing the boundary between its own chromosome and the other surrounding chromosome territories. As discussed previously, there are no known sequence determinants of this recognition and the factors that prevent *XIST* from silencing *in trans* are not currently understood. We speculate this is related to *cis* attachment to an underlying scaffold of *XIST*'s parent chromosome territory.

## 4. *XIST* RNA and the debated concept of a complex non-chromatin nuclear matrix

Perhaps in agreement with the above idea (§3), the list of potential *XIST*-interacting proteins generated recently included a number of SAF-A related proteins that could influence how *XIST* interacts with the chromosome [8–10]. Several of these proteins, including SAF-A, are characterized as being constituents of the insoluble nuclear scaffold (also known as the nuclear matrix). And others, such as the lamin B receptor (LBR) are attached to nuclear lamina structure [7]. The nuclear matrix is defined as the insoluble, non-chromatin material remaining after extensive biochemical fractionation which removes chromatin, including 90–95% of DNA, histones and most nuclear proteins [47–49]. Furthermore, approximately 70% of heterogeneous nuclear RNA (much of which does not encode protein) was reported to remain bound to the nuclear scaffold. Whether the remaining ‘fibrillogranular’ ultrastructure, visualized by electron microscopy, constitutes a bona fide *in vivo* structure had earlier been the subject of intense controversy and is still not fully resolved despite extensive literature on the subject [50,51]

**Figure 2.**
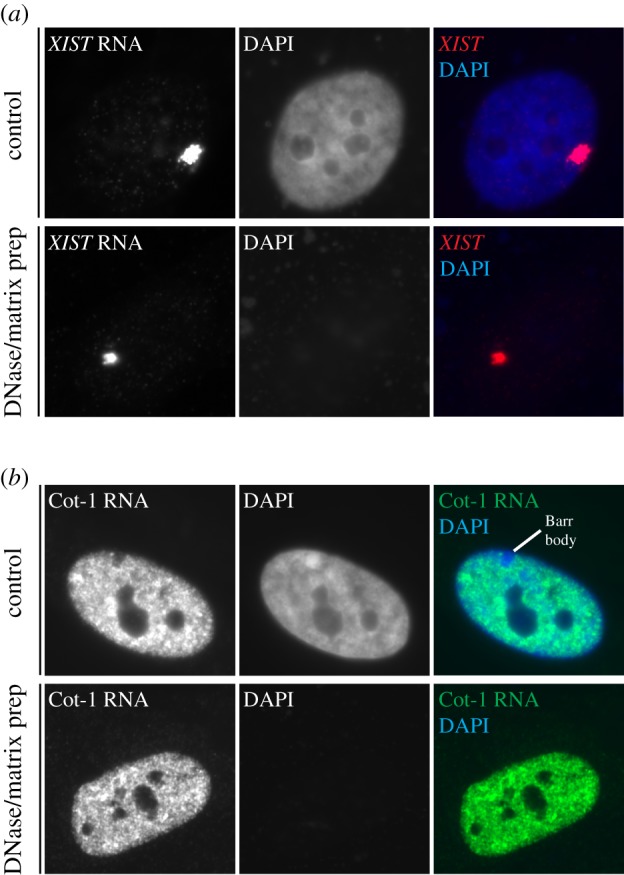
Evidence that *XIST* RNA and repeat-rich RNAs are bound with non-chromatin nuclear scaffolding. (*a*) RNA FISH experiments demonstrating that *XIST* RNA (red) remains in a discrete RNA chromosome territory in TIG1 human fibroblast in control treated cells (top) or after removal of DNA (blue) by DNAse digestion and extraction of histones and most other nuclear proteins, using previously described nuclear matrix fractionation procedures. *XIST* RNA remains essentially unperturbed in a bright localized nuclear territory with the small fraction of proteins that remain insoluble, including SAF-A (territory sizes vary between cells but are similar in both conditions). (*b*) Cot-1 RNA detected by RNA FISH in human TIG1 fibroblasts. The bright signal distributes over most of the DNA (blue, DAPI) signal, but is absent from large regions of DNA-dense heterochromatin such as the Barr body. Like *XIST*, Cot-1 RNA is unperturbed by extraction of soluble proteins and digestion of chromatin, suggesting repeat-rich RNAs are also embedded in the insoluble nuclear scaffold.

Against this background, a key and surprising early finding for us was that the bright nuclear *XIST* RNA territory remains tightly defined and unperturbed in a nuclear matrix preparation after removal of chromosomal DNA (figure 2*a*) [52]. This seemed paradoxical for an RNA which at the same time we argued had an unprecedented adherence to interphase chromosome structure. This led us early on to propose a model for complex structural interactions between *XIST* RNA, canonical chromatin (DNA packaged with histones), and the putative nuclear matrix/scaffold. The evidence now that *XIST* RNA interacts specifically with SAF-A and other proteins related to nuclear structure fits and supports this model, underpinning our perspective that *XIST* RNA is not simply ‘tethered’ to the chromatin, but structurally intertwined not only with SAF-A, but what we envision as a complex lattice of scaffolding proteins. Many apparent *XIST*-interacting proteins are widely distributed and abundant constituents of the nuclear matrix, which we envision interact in a complex, mutually dependent meshwork with which *XIST* RNA is not just tethered, but structurally embedded.

The results from Kolpa *et al*. [45] demonstrate that strict reliance on SAF-A for anchoring *XIST* RNA to Xi seems to occur only in certain transformed cell lines and pluripotent stem cells. While *XIST* localization in cancer cells and stem cells is clearly important, the majority of normal differentiated cells appear to have redundant or complex mechanisms to faithfully anchor *XIST* to Xi. Interestingly, irregular nuclear shape and compromised nuclear scaffolding has been observed in transformed and cancer cells [53,54]. In fact, *XIST* is functionally compromised or mislocalized and the Barr body is not observable in some cancers [55,56]. Therefore, we suggest that immortalized cells may have a ‘weakened’ nuclear matrix and looser embedment of *XIST* RNA. Similarly, pluripotent stem cells, which also require SAF-A for *XIST* expression/localization, have less developed nuclear substructure and express very low levels of the nuclear matrix protein lamin A/C [57,58]. We speculate this also sensitizes pluripotent cells to loss of SAF-A.

Finally, we note that SAF-A and other nuclear matrix proteins, including many reported to associate with *XIST* RNA, are not restricted to the Xi, but are distributed widely over chromatin. Thus, it is plausible that this repertoire of scaffolding proteins could interact with other nuclear non-coding RNAs to modify chromatin architecture throughout the genome [59,60]. This perspective, together with our interest in ‘junk’ of the genome, led us to explore the possibility that *XIST* RNA exemplifies a broader paradigm for non-coding RNA function in nuclear organization.

## 5. Repeat-rich RNA is embedded in nuclear ‘scaffolding’

Remarkably, abundant repeats including LINEs, SINEs, transposable elements and simple sequences, comprise over half the human genome, but their potential contribution to chromosome structure and regulation remains under studied. Rather than avoid analysis of repetitive sequences, our recent work intentionally targeted analysis of ‘junk’ that comprises the bulk of chromosomes. Using Cot-1 DNA (the repeat-rich and rapidly annealing fraction of the genome after fragmentation) as a probe in RNA FISH experiments, we identified a remarkably abundant and stable association of repeat RNA with the interphase chromosome territory of active chromosomes in all human and mouse cell types tested [61]. Cot-1 RNA is present and localized *in cis* even after long-term inhibition of transcription and is unperturbed by high salt extraction and DNase digestion of chromatin (figure 2*b*), suggesting that like *XIST*, Cot-1 RNA is in tight association with a nuclear scaffold. Consistent with this idea, Cot-1 RNA signal is mislocalized in cells expressing dominant-negative mutants of SAF-A [61]. If and how SAF-A more generally complexes RNA with chromatin will be the subject of future study.

Cot-1 RNA, though broadly distributed in nuclei of all cell types tested, is conspicuously absent over constitutive and facultative heterochromatin (such as the nuclear periphery and the Barr body, see figure 2*b*), clearly indicating a preferential association with euchromatic regions. Cot-1 RNA is released from mitotic chromosomes (much like *XIST*) and resynthesized in G1 daughter cells after cell division. Inhibiting the resynthesis of these RNAs after cell division with transcriptional inhibitors prevents the reorganization and ‘opening up’ of nuclei. These, and other results suggest that Cot-1 RNA may be required to promote an active chromatin state. In support of this hypothesis, RNase treatment of nuclei is known to cause rapid ‘collapse’ of chromatin structure as judged by DAPI staining [61–63]. The immediacy of this effect suggests that rather than directing histone modifications, RNA can act at the structural level to impact higher-order organization of chromatin. Since Cot-1 RNA is notably absent from the chromosome territory of the inactive X chromosome and in light of an apparent property of RNAs to keep chromatin ‘open’, we further speculate that *XIST* functions somehow to strip other RNAs or reorganize chromatin in a way that contributes to cytologically observable compaction of the Barr body.

How exactly might repeat-rich ‘junk’ RNA influence this architecture? We consider that the presence of nascent transcripts themselves may open chromatin. If this is the case, the prevalence of repeat sequences in introns may prove significant. While it is not widely appreciated, the distribution of repeat families in the genome is non-random and has been proposed to have important implications for regulating gene expression, as we have discussed in greater depth recently [59]. This may be, in part, because DNA sequences of repeats are rich in protein binding sites, sometimes referred to as repeat-associated binding sites (RABS) [64,65]. RABS can make up a substantial portion of known transcription factor binding sites, with individual repeats or repeat families conferring specificity of binding. Thus it appears that repeat sequences can influence our epigenome, contributing to the evolution of promoters and enhancers that regulate gene expression. In addition, repeat sequences have increased capacity to form secondary structures such as G-quadruplex and RNA/DNA triplexes, adding to the diversity of potential interactions mediated by repetitive sequences in RNA [59,66,67].

We have only begun to explore the potential for functional repeats in nuclear RNA. Recently, it has been demonstrated that a substantial portion of mammalian proteomes bind RNA, many through poorly understood low-complexity domains [68]. Much as the transcription factor binding motifs have been annotated over the past decades, we speculate that as the sequence specificity of RNA binding proteins is determined, repeats will play an important role in conferring this specificity as well. Repetitive sequences could then serve as common binding sites for proteins that connect into the nuclear matrix, impacting local chromatin architecture or perhaps acting as a platform for binding to chromatin, chromatin modifying complexes, or the transcriptional apparatus itself.

To test these hypotheses we need to learn more about the specificity of RNAs remaining in the nuclear scaffold fraction. Chromatin-associated RNAs have recently been catalogued [69]. Future genomic experiments should directly test what RNAs are embedded in the non-chromatin nuclear scaffold and if they differ from chromatin-associated RNAs.

## 6. RNA, a fundamental component of interphase chromosomes?

**Figure 3.**
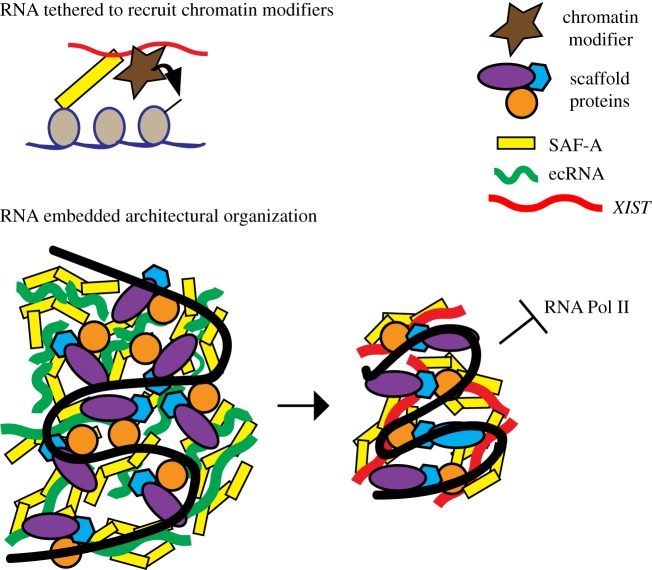
Distinct models for *XIST* RNA chromosomal interactions and function. Top: XIST *RNA is tethered to the chromosome to recruit chromatin modifiers*. This model incorporates the suggestion that *XIST* RNA is tethered by a single molecule bridge which directly connects RNA to chromosomal DNA (adapted from Hasegawa *et al*. [41]; see also Kolpa *et al*. [45]), and other evidence that *XIST* RNA functions by recruiting histone modifying enzymes to modify chromatin. In this model, the RNA need not be intertwined with other chromosome structural elements. Bottom: *Chromosomal RNAs may serve as an embedded component of chromosome structure, with different types of RNA impacting the arrangement of non-chromatin architectural elements*. This model schematically depicts the potential for different classes of chromosome associated RNAs to interact with similar structural elements, but influence their arrangement in distinct ways to promote open euchromatin (left) versus more dense heterochromatin (right). A chromosomal domain is represented and intended to illustrate that RNA may localize and act via classic components of the nuclear matrix (SAF-A, lamins, matrins, NUMA and other hnRNPs) which form a complex interconnected meshwork. DNA binding proteins may act as a bridge between the scaffold and chromatin (DNA packaged with canonical histones, shown in black). Left shows euchromatin-associated RNAs (ecRNAs) such as repeat-rich Cot-1 RNA. Right shows a chromosomal domain with *XIST* RNA. Both interact with similar scaffold proteins but with distinct higher-order organization.

Increasingly, non-coding RNAs are being identified which regulate usage of chromatin. It is reasonable that *XIST* RNA will serve as a window into understanding how many other non-coding RNAs, whether promoting or repressing transcription, interface with chromosomes. As discussed in §4 for *XIST*, much emphasis has been on the potential of RNA to modify epigenetic state via recruitment of histone modifications. For this role, it could be sufficient to tether the RNA to chromatin by a single binding partner (figure 3, top). However, based on findings highlighted here, we postulate that RNA is more embedded in nuclear structure, and may serve essentially as an architectural element of the chromosome (figure 3, bottom). In this model, rather than a protein such as SAF-A serving to localize *XIST* RNA, we hypothesize that *XIST* RNA may actually act on or via SAF-A, or similar ‘architectural’ proteins, in a manner that impacts their arrangement, thereby more directly modifying higher-order chromatin structure. As schematically shown in figure 3, RNAs associated with heterochromatin versus euchromatin could interact with the same or similar scaffold proteins, but modify their architectural arrangement in a distinct way, thereby impacting large-scale packaging (e.g. condensation) across a chromosomal domain. A structural contribution of RNA to nuclear chromatin is also suggested by the rapid condensation of DNA following RNAse, an impact clearly independent of canonical chromatin modifications.

This model does not preclude that *XIST* or other RNAs recruit chromatin modifying enzymes; however, several basics of *XIST* RNA cell biology support a working model which includes the RNA's potential as a ‘structural’ element. These observations include: (i) the retention of *XIST* RNA in a bright, localized nuclear territory even after removal of chromatin [52], (ii) the involvement of SAF-A, which is well-characterized as a structural component of the ‘insoluble non-chromatin nuclear scaffold’ [42], (iii) the greater impact of certain SAF-A mutants on *XIST* RNA localization in contrast to mild effects of SAF-A knockdown, consistent with dominant negative effects on other elements of the inter-connected scaffold [45], and (iv) the fact that *XIST* RNA does not require chromosome-specific sequences, yet clearly and comprehensively recognizes the structure of the chromosome from which it is transcribed [26].

Whatever the detailed mechanisms, it is intuitive that changes in condensation can be propagated across a large chromosome, and this likely involves repeating patterns of higher-order folding. Thus, interspersed repetitive sequences could well be involved in propagation of chromatin state. In addition, a recently recognized property of many RNA binding proteins suggests to us an additional concept of how they might propagate chromatin architecture. RNA binding proteins, including SAF-A, disproportionately contain low-complexity domains of polar, uncharged amino acids predicted to be ‘prion-like’ [70]. Prion-like domains can promote aggregation and are known in some cases to facilitate self-templating fibrilization, mediate liquid-phase transitions, and in some cases form so-called ‘membraneless organelles’, as reviewed recently [70]. Recent findings indicate this is intriguingly relevant to the role of SAF-A, based on efforts from our laboratory and that of Nakagawa to resolve a difference between our findings. We were surprised when we could not confirm the requirement for the RGG domain of SAF-A, previously suggested to be responsible for *XIST*-binding [41], to localize *XIST* RNA. We speculate this may have to do with the slightly larger deletion used in this study. Sakaguchi and colleagues then tested additional mutants, and, collectively, these studies indicate that the C-terminal region of the SAF-A prion-like domain, even lacking the RGG motifs, can still largely support *XIST* RNA association with chromosome structure [46]. Since recombinant SAF-A/DNA complexes were long-ago shown to form visually stunning filamentous multimers by electron microscopy [38], it will now be compelling to consider a model whereby SAF-A can influence long-range chromosome architecture via aggregation. Does *XIST* RNA modify these interactions? Since SAF-A (and proteins like it) are widely distributed within mammalian nuclei, do these interact with other non-coding RNAs (e.g. repeat-rich RNAs), perhaps in a manner that differs from *XIST* RNA?

## 7. Concluding remarks

The model forwarded here is clearly influenced by the concept of an insoluble, non-chromatin nuclear scaffolding, also termed the nuclear matrix, which we recognize remains somewhat controversial or not fully established. However, this is a fundamentally important concept with profound implications for understanding epigenome regulation, since chromatin-associated RNA may act with nuclear scaffolding to modify chromatin architecture. We assert that properties of *XIST* RNA described above strongly support the *in vivo* reality of some form of non-chromatin nuclear scaffold/matrix. However, since early studies reported that as much as 70% of nuclear RNA remains with the matrix fraction, a long-standing criticism has been that the extensive extraction procedures can precipitate RNAs on a proteinaceous residue. Hence, it will be important to use genomic approaches and nuclear fractionation techniques to establish whether RNAs isolated from the nuclear scaffold fraction represent a random precipitate or indeed a distinct population including a fundamentally important subset of long non-coding RNAs that are embedded with interphase chromosome structure.

Currently, the mechanisms of how chromosomal, or ‘architectural’, RNAs could then contribute to regulation of gene expression are unclear. As described here and elsewhere, even for *XIST*, undoubtedly the principal paradigm for negative regulation of transcription by a non-coding RNA, details remain elusive or controversial; to our knowledge, no experiment or model system has definitively demonstrated the relative importance of the myriad of semi-redundant activities (figure 1*a*) downstream of *XIST* in repressing transcription. One of these mechanisms clearly seems to be recruitment of histone modifying enzymes. While control of individual gene transcription can be under ‘local’ control (e.g. histone modification at promoters), regulation of larger chromosomal regions may involve creation of nuclear domains, similar to the localization of inactive genes in the peripheral heterochromatic compartment [71] or adjacent to heterochromatic chromocentres [72]. *XIST* RNA triggers (directly or indirectly) global architectural reorganization to form a silent nuclear compartment lacking Cot-1 RNAs and RNA polymerase II. Association of genes with this domain (directly abutting it or within the periphery) could contribute to their robust silencing, as we have discussed elsewhere [19]. We speculate that some nuclear RNAs, including *XIST*, may modulate their surrounding environment to influence transcription through directly enacting architectural changes by embedding with and/or modifying RNA binding proteins in the nuclear scaffold that regulate the accessibility or utilization of nearby chromatin.

## Data accessibility

This article has no additional data.
